# Evidence of Air and Surface Contamination with SARS-CoV-2 in a Major Hospital in Portugal

**DOI:** 10.3390/ijerph19010525

**Published:** 2022-01-04

**Authors:** Priscilla Gomes da Silva, José Gonçalves, Ariana Isabel Brito Lopes, Nury Alves Esteves, Gustavo Emanuel Enes Bamba, Maria São José Nascimento, Pedro T. B. S. Branco, Ruben R. G. Soares, Sofia I. V. Sousa, João R. Mesquita

**Affiliations:** 1 ICBAS–School of Medicine and Biomedical Sciences, Porto University, 4050-313 Porto, Portugal; up202002072@edu.icbas.up.pt; 2 Epidemiology Research Unit (EPIunit), Institute of Public Health, University of Porto, 4050-600 Porto, Portugal; 3 LEPABE–Laboratory for Process Engineering, Environment, Biotechnology and Energy, Faculty of Engineering, University of Porto, 4200-465 Porto, Portugal; up200606911@fe.up.pt (P.T.B.S.B.); sofia.sousa@fe.up.pt (S.I.V.S.); 4 Institute of Sustainable Processes, University of Valladolid, 47011 Valladolid, Spain; zemcg5@gmail.com; 5 Department of Chemical Engineering, University of Valladolid, 47011 Valladolid, Spain; 6 Unidade Local de Saúde do Alto Minho E.P.E., 4904-858 Viana do Castelo, Portugal; ariana.lopes@ulsam.min-saude.pt (A.I.B.L.); nury.esteves@ulsam.min-saude.pt (N.A.E.); gustavo.bamba@ulsam.min-saude.pt (G.E.E.B.); 7 Faculty of Pharmacy, University of Porto, 4050-313 Porto, Portugal; saojose@ff.up.pt; 8 Department of Biochemistry and Biophysics, Science for Life Laboratory, Stockholm University, SE-106 91 Stockholm, Sweden; ruben.soares@scilifelab.se

**Keywords:** SARS-CoV-2, environmental contamination, air samples, surface samples

## Abstract

As the third wave of the COVID-19 pandemic hit Portugal, it forced the country to reintroduce lockdown measures due to hospitals reaching their full capacities. Under these circumstances, environmental contamination by SARS-CoV-2 in different areas of one of Portugal’s major Hospitals was assessed between 21 January and 11 February 2021. Air samples (*n* = 44) were collected from eleven different areas of the Hospital (four COVID-19 and seven non-COVID-19 areas) using Coriolis^®^ μ and Coriolis^®^ Compact cyclone air sampling devices. Surface sampling was also performed (*n* = 17) on four areas (one COVID-19 and three non-COVID-19 areas). RNA extraction followed by a one-step RT-qPCR adapted for quantitative purposes were performed. Of the 44 air samples, two were positive for SARS-CoV-2 RNA (6575 copies/m^3^ and 6662.5 copies/m^3^, respectively). Of the 17 surface samples, three were positive for SARS-CoV-2 RNA (200.6 copies/cm^2^, 179.2 copies/cm^2^, and 201.7 copies/cm^2^, respectively). SARS-CoV-2 environmental contamination was found both in air and on surfaces in both COVID-19 and non-COVID-19 areas. Moreover, our results suggest that longer collection sessions are needed to detect point contaminations. This reinforces the need to remain cautious at all times, not only when in close contact with infected individuals. Hand hygiene and other standard transmission-prevention guidelines should be continuously followed to avoid nosocomial COVID-19.

## 1. Introduction

SARS-CoV-2 infection causes respiratory illness ranging from mild to severe disease and death, with some infected people being asymptomatic [1]. According to the World Health Organization (WHO), evidence shows that SARS-CoV-2 spreads mainly between people who are standing near one another. In these situations, aerosols or droplets produced by an infected person and which contain the virus are inhaled or come directly into contact with the nose or mouth of a susceptible person, particularly in poorly-ventilated and crowded indoor environments [2].

Respiratory droplets formed from respiratory secretions and saliva are emitted through talking, coughing, sneezing and even breathing, and have a diameter ranging from <1 µm to >100 µm [3]. Respiratory droplets are generally defined as particles that fall to the ground (or any surface) more quickly under the influence of gravity due to their larger size; typically, these are particles > 5–10 µm that fall within 2 m of the source. When these particles settle on surfaces, the contaminated surfaces are then called fomites. Once emitted by humans, these respiratory droplets tend to reduce in size due to evaporation, after which they are termed as droplet nuclei or aerosols, which can be defined as particles that remain suspended due to size and/or environmental conditions; typically, these are particles ≤ 5 µm that stay suspended in air for longer, eventually falling to the ground if the air is motionless for long enough (at least 30 min) [3]. Considering that aerosols are small enough to remain suspended in air, they can accumulate in poorly-ventilated spaces and in turn be inhaled at both short and long ranges by a susceptible person, indicating the importance of improving and ensuring good indoor ventilation in the context of COVID-19 [4]. Moreover, the produced aerosols contain much higher viral loads when compared to viral loads in droplets [5,6,7,8]. Hence, proper ventilation can reduce surface contamination by removing virus particles before they can land on surfaces [9].

Despite droplet and fomite transmissions being considered the probable main modes of transmission for SARS-CoV-2, these alone cannot account for superspreading events [10,11,12], or for differences in transmission between indoor and outdoor environments [8]. SARS-CoV-2 modes of transmission are now distinguished as inhalation of virus, deposition of virus on exposed mucous membranes, and touching mucous membranes with soiled hands contaminated with virus [13]. However, individuals who come into contact with potentially infectious surfaces or aerosols containing viral particles often have close contact with an infected person, making it difficult to distinguish the source of infection as being airborne or through fomites [14], particularly in healthcare institutions where infected individuals continuously excrete high viral loads into the environment, potentially adding to the occupational risk of healthcare professionals [15]. In fact, while the risk of transmission via environmental contamination of SARS-CoV-2 is considered to be generally low [16], a number of factors may increase this risk, particularly considering hospital environments where a high number of symptomatic patients with active infection and increased viral shedding might be present when compared to the situation in the community outside the hospital environment [17].

At the beginning of this study, 21 January 2021, there were 151,226 active cases of COVID-19 in Portugal and 702 people admitted in intensive care units (ICUs) all over the country [18]. To the best of the authors’ knowledge, no study on environmental contamination with SARS-CoV-2 in hospital settings has been performed in Portugal; hence, this study aimed to assess air and surface contamination in different areas of a major Hospital in Portugal during the peak of the third wave of COVID-19 in the country (late December 2020 to mid-February 2021). The study also aimed at assessing the performance and suitability of two air samplers (Coriolis^®^ μ and Coriolis^®^ Compact) for SARS-CoV-2 air monitoring. The results may be relevant in establishing interventions to prevent healthcare workers’ exposure to SARS-CoV-2 and to optimize and better understand the extent of environmental viral contamination of surfaces in healthcare settings.

## 2. Materials and Methods

**Sampling sites**

Environmental sampling took place in a Hospital in Portugal that serves around 2.2% of the Portuguese population, between 21 January and 11 February 2021. Air samples (*n* = 44) were collected from four COVID-19 and seven non-COVID-19 areas. COVID-19 areas included the COVID-19 ICU, intermediate COVID-19 ICU, COVID-19 nursing area and the COVID-19 testing room. Non-COVID-19 areas included the respiratory diseases observation room, respiratory diseases waiting room, clinical decision unit, non-respiratory diseases patients’ waiting room, urgent care (recovery area), the Hospital’s outside entrance atrium and the Hospital’s staff cafeteria.

Surface sampling (*n* = 17) was performed on four areas, of which three were non-COVID-19 areas (non-respiratory disease waiting room, staff cafeteria, and outside entrance atrium) and one COVID-19 area (the COVID-19 testing room). Further details about each sampling site are summarized in Table 1 and Table 2.

**Collection of air and surface samples**

Air samples were collected using two cyclonic microbial air samplers, a Coriolis^®^ μ and a Coriolis^®^ Compact (Bertin Instruments, Montigny-le-Bretonneux, France). Using the Coriolis^®^ μ, three consecutive air samplings were collected from each of the eleven areas of the Hospital for 10 min each with an airflow rate of 100 L/min (total of 1 m^3^), 200 L/min (total of 2 m^3^) and 300 L/min (total of 3 m^3^), respectively. Air samples with the Coriolis^®^ μ were collected on wet medium, with 4 mL of sterile phosphate buffered saline (PBS) added to the collection cones before sampling. With the Coriolis^®^ Compact, one air sampling was performed in the same eleven areas for 60 min, with an airflow rate of 50 L/min (total of 3 m^3^). Air samples with Coriolis^®^ Compact were collected on dry medium, with 4 mL of sterile PBS added to the collection cones after sampling. Both Coriolis^®^ samplers were placed side by side at 1.3 m height using a portable table, and the three consecutive Coriolis^®^ μ samplings were performed simultaneously within the 60-min sampling periods of the Coriolis^®^ Compact.

Surface samples were collected on 10 cm × 10 cm surface areas (100 cm^2^ area per sampling) using sterile flocked plastic swabs previously wetted on PBS and immediately placed in PBS (4 mL).

All samples were stored at 4 °C before being taken to the laboratory facilities, and were processed within 8 h. Details about the characteristics of the air and surface samples collected are summarized in Table 2 and Table 3, respectively.

**RNA extraction and detection of SARS-CoV-2**

RNA extraction was performed using the GRS Viral DNA/RNA Purification Kit (GRISP, Porto, Portugal) according to the manufacturer’s instructions. RNA extraction was performed on 200 μL of sample suspensions as previously described [19]. A one-step RT-qPCR reaction aimed at two viral gene targets (N1 and N2) using viral target-specific primers and Taqman probe technology based on a previously described protocol [20] was used (Xpert qDetect COVID-19, GRISP, Porto, Portugal). For the CFX Real-Time PCR (qPCR) Detection System (Bio-Rad, Hercules, CA, USA), the Bio-Rad CFX Maestro 1.0 Software version 4.0.2325.0418 was used to control the runs and remotely analyze the data. Each RT-qPCR run included ssDNA targets for both N1 and N2 regions (positive controls) and a no-template control. Reactions were set up and run with initial conditions of 15 min at 45 °C and 2 min at 95 °C, then 45 cycles of 95 °C for 15 sec and 55 °C for 30 sec. A standard curve was constructed using the ssDNA targets for both N1 and N2 regions in a 10-fold serial dilution mixture starting at 200,000 copies/µL, in order to quantify the number of viral gene copies present in each sample from the measured Ct values; the limit of detection (LOD) was 1.3 copies/µL for N1 and 3.2 copies/µL for N2. Air sample results are expressed in copies/m^3^, and surface sample results in copies/cm2.

## 3. Results

Of the 44 air samples collected in eleven different areas of the Hospital, only two (C1 and M1) were positive for SARS-CoV-2 RNA (Table 4). They were both from the same place, the COVID-19 ICU, and were collected at the same time; C1 (viral loads of 6000 and 6575 copies/m^3^ for N1 and N2 genes, respectively) was collected during 60 min sampling with the Coriolis^®^ Compact at an airflow rate of 50 L/min (total of 3 m^3^), while M1 (viral loads of 6362.5 and 6662.5 copies/m^3^ for N1 and N2 genes, respectively) was collected with the Coriolis^®^ μ during the first 10 min of the Coriolis^®^ Compact collection period, at an airflow rate of 100 L/min (total of 1 m^3^). The two other Coriolis^®^ μ consecutive samples (M2 and M3) collected within the 60-min time frame of the Coriolis^®^ Compact (air flow rates of 200 L/min and 300 L/min, respectively) were both negative for SARS-CoV-2 RNA.

Of the 17 surface samples collected in four different areas of the Hospital, three were positive for SARS-CoV-2 RNA, with viral loads of 200.6 copies/cm^2^ (COVID-19 testing room, wall of a testing booth), 179.2 copies/cm^2^ (non-respiratory disease waiting room, faucet handle), and 201.7 copies/cm^2^ (non-respiratory disease waiting room, bathroom’s flush button). The three samples amplified only one of the two target genes (N1, N1 and N2 respectively). Details on the Hospital area, sampling location and viral genome copy numbers of the positive air and surface samples are summarized in Table 4.

## 4. Discussion

The present study aimed to evaluate SARS-CoV-2 environmental contamination of air and surfaces in a major Hospital in Portugal during the third wave of the COVID-19 pandemic. Eleven different areas of the Hospital were selected to be assessed for air contamination, including four COVID-19 and seven non-COVID-19 areas. SARS-CoV-2 RNA was only detected in the air of the COVID-19 ICU. The viral load of these air samples collected with the Coriolis^®^ Compact and Coriolis^®^ μ ranged from 6000 to 6662.5 copies/m^3^. Interestingly, only the first sample of Coriolis^®^ μ, collected during the first 10 min of the 60-min time frame of the Coriolis^®^ Compact, was SARS-CoV-2 RNA positive. The two other consecutive samplings of Coriolis^®^ μ performed within the 60-min time frame of the Coriolis^®^ Compact were negative for SARS-CoV-2 RNA in spite of a higher airflow rate (air flow rates of 200 L/min and 300 L/min, respectively), suggesting a point contamination which could be explained by the fact that, when the sampling period started, a patient had just been intubated. This intubation can explain the presence of aerosols containing virus during the first 10 min of Coriolis^®^ μ, sampling and the negative results in the second and third samples, considering that aerosols containing SARS-CoV-2 may have been removed by the rooms’ ventilation system by the time the other two samplings took place.

In this study, we aimed to assess the performance and suitability of both air samplers for SARS-CoV-2 air monitoring. The results of this study suggest that Coriolis^®^ µ and Coriolis^®^ Compact samplers seem robust for SARS-CoV-2 air sampling, as both were able to detect SARS-CoV-2 RNA in the air of the COVID-19 ICU. However, longer collection times are more likely to cover point contaminations, as was seen with the 60-min collection with Coriolis^®^ Compact. During this collection, three consecutive samplings of Coriolis^®^ μ were performed, with only the first providing a positive air sample.

To assess contamination of surfaces, four different areas of the Hospital were selected, one COVID-19 area and three non-COVID-19 areas. SARS-CoV-2 RNA was detected on the wall of one of the testing booths (where patients wait for nasopharyngeal sample collection), which was somewhat expected due to the fact that during sampling there were possibly-infected individuals constantly coming in to collect nasopharyngeal samples for testing, all of them reporting as symptomatic (presenting respiratory symptoms such as coughing or sneezing). Moreover, this is the only room where patients remove their masks, likely increasing the viral load in indoor air. Contamination in this room might have happened through respiratory aerosols or droplets from the infected patients that settled on the surfaces sampled, considering that neither the patients or healthcare staff touch the walls in this area, ruling out the possibility of direct touch contamination in this case. SARS-CoV-2 viral RNA was detected in the non-respiratory disease waiting room as well. This was one of the non-COVID-19 areas sampled, and therefore the presence of viral RNA was not expected. This was a positive pressure room, which ideally prevents unfiltered air from outside the room from coming inside [21]. Nevertheless, viral RNA was detected in two surface samples in this area, namely on a faucet handle located in the middle of the waiting room and on the flush button of a bathroom in this area. As these are frequently-touched surfaces, the most likely explanation for viral RNA presence is direct touch contamination. Nonetheless, SARS-CoV-2 excretion in stools has been described, and the flushing could generate contaminated aerosols that could ultimately deposit on these surfaces [22,23]. These results highlight the importance of hand and general hygiene in public toilets as well as the need for enhanced disinfection protocols in all areas of the Hospital, not only those dedicated to COVID-19 patients.

Airborne transmission of SARS-CoV-2 is now widely accepted as a mode of transmission of SARS-CoV-2 [2,4,8,12]. This route dominates under certain environmental conditions, particularly indoor environments without proper ventilation [24,25,26,27,28,29], with a recent study demonstrating that some fraction of the RNA-containing aerosols emitted from infected people contain intact, replication-competent virions [30]. On the contrary, current evidence suggests that transmission through contaminated surfaces is rare; however, when it comes to healthcare settings where COVID-19 patients are being treated, especially in ICUs, aerosol-generating medical procedures take place which could potentially exacerbate the contamination of air and surfaces in the surrounding area [31]. To avoid nosocomial infection of healthcare workers, non-COVID-19 patients, and visitors by these virus-laden aerosols as well as to avoid contamination of medical equipment and surfaces, it is imperative that hospital ventilation works properly [4].

COVID-19 patients that need to undergo intubation and extubation are usually placed in negative-pressure isolation rooms, which is considered to be safer [32]. Negative-pressure rooms have a ventilation system in which air flows from the exterior to the interior [21]. This keeps aerosolized viruses from spreading through the heating, ventilation, and air conditioning system. Under these conditions, if an opening exists, air will flow from the surrounding areas into the negatively pressurized space [4]. In the Hospital assessed in this study, the COVID-19 ICU, which was the only negative room sampled, had twelve air changes per hour (ACH), in compliance with the safety rules for these types of rooms [31].

Since the beginning of the pandemic, many articles have been published on detection of SARS-CoV-2 in air samples [24,25,33,34,35]; however, some inherent problems with air sampling for the detection of viruses have come up, such as the limited diversity of monitored spaces (most studies are done in indoor healthcare facilities), limited number of samples, diversity of methodologies (there is no gold-standard protocol for air sampling of SARS-CoV-2 and other airborne viruses), not all studies performing both surface and air sampling simultaneously, and lastly, the fact that most of these studies were performed during the first wave when little was known about the virus, which could have led to errors in methods and molecular analysis.

This study faced some limitations that are worth highlighting. This was an observational study at a single hospital, which means that the results may not be generalizable to other healthcare facilities. Additionally, no assessment of virus viability was performed, as no BSL3 facility was available to perform such experiments. As a result, the findings in this study, although reflecting the real extent of environmental contamination with SARS-CoV-2 in the Hospital, do not necessarily amount to an infection risk assessment for air and surfaces. Moreover, no surface sampling was performed in the COVID-19 ICU, and we do not have individual data on patients, particularly those who occupied the COVID-19 ICU at the time of sampling. Patients with severe infection influence the viral load in droplets and exhaled aerosols; therefore, it is important that in future studies these individual patient data are acquired in order to allow better interpretation of results. Nevertheless, this study is an important addition to the growing literature on the detection of SARS-CoV-2 RNA in air and on surfaces.

## 5. Conclusions

The present study showed SARS-CoV-2 hospital environmental contamination both in air and on surfaces in locations where both COVID-19 and non-COVID-19 patients were present. This reinforces the need to remain cautious at all times, not only when in close contact with infected individuals. Hand hygiene and other standard transmission-prevention guidelines should be continuously followed in order to avoid nosocomial COVID-19. Further studies combining air and surface sampling with virus viability assays are still needed to fully elucidate the real risk of air and environmental transmission in healthcare facilities.

## Figures and Tables

**Table 1:** Air ventilation details about the sampling sites.

	Hospital Area	Type of Ventilation and Pressure	People with Access to This Area
COVID-19 areas	COVID-19 ICU	Mechanic ventilation, negative pressure	Patients and Hospital staff
	Intermediate COVID-19 ICU	Mechanic ventilation, negative pressure	Patients and Hospital staff
	COVID-19 nursing area	Mechanic ventilation, negative pressure	Patients and Hospital staff
	COVID-19 testing room	Mechanic ventilation, negative pressure	Patients and Hospital staff
Non-COVID-19 areas	Respiratory diseases observation room	Natural ventilation, neutral pressure	Patients and Hospital staff
	Respiratory diseases waiting room	Natural ventilation, neutral pressure	Patients, Hospital staff and patients’ companions
	Non-respiratory diseases waiting room	Natural ventilation, neutral pressure	Patients, Hospital staff and patients’ companions
	Clinical decision unit	Natural ventilation, neutral pressure	Patients and Hospital staff
	Urgency care (recovery area)	Natural ventilation, neutral pressure	Patients and Hospital staff
	Hospital’s outside entrance atrium	Natural ventilation, neutral pressure	Open to the general public
	Hospital staff’s cafeteria	Natural ventilation, neutral pressure	Hospital staff

**Table 2:** Details of the air samples’ collections for SARS-CoV-2 RNA detection in the Hospital.

Device	Sample ID	Date of Collection	Hospital Area	Sampler Location	Sampling Parameters
Coriolis^®^ Compact	C1	21 January 2021	ICU COVID-19	Air sampler placed approximately 1.3 m above the floor and 2 m from a patient bed, in the center of the room	50 L/min, 60 min
	C2	21 January 2021	ICU intermediate COVID-19	Air sampler placed approximately 1.3 m above the floor and 2 m from a patient bed, in the center of the room	50 L/min, 60 min
	C3	21 January 2021	Nursing area COVID-19	Air sampler placed approximately 1.3 m above the floor and 2 m from a patient bed, in the center of the room	50 L/min, 60 min
	C4	27 January 2021	Respiratory diseases observation room	Air sampler placed approximately 1.3 m above the floor and 2 m from a patient bed, in the center of the room	50 L/min, 60 min
	C5	27 January 2021	Respiratory diseases waiting room	Air sampler placed approximately 1.3 m above the floor in the center of the room	50 L/min, 60 min
	C6	27 January 2021	Clinical decision unit	Air sampler placed approximately 1.3 m above the floor and 2 m from a patient bed, in the center of the room	50 L/min, 60 min
	C7	2 February 2021	COVID-19 testing room	Air sampler placed approximately 1.3 m above the floor in the center of the room	50 L/min, 60 min
	C8	2 February 2021	Non-respiratory diseases waiting room	Air sampler placed approximately 1.3 m above the floor in the center of the room	50 L/min, 60 min
	C9	2 February 2021	Urgency Care (Recovery area)	Air sampler placed approximately 1.3 m above the floor and 2 m from a patient bed, in the center of the room	50 L/min, 60 min
	C10	11 February 2021	Hospital’s outside entrance atrium	Air sampler placed approximately 1.3 m above the floor in the center of the room	50 L/min, 60 min
	C11	11 February 2021	Hospital employe’s cafeteria	Air sampler placed approximately 1.3 m above the floor in the center of the room	50 L/min, 60 min
Coriolis^®^ μ	M1	21 January 2021	ICU COVID-19	Air sampler placed approximately 1.3 m above the floor and 2 m from a patient bed, in the center of the room	100 L/min, 10 min
	M2	21 January 2021	ICU COVID-19	Air sampler placed approximately 1.3 m above the floor and 2 m from a patient bed, in the center of the room	200 L/min, 10 min
	M3	21 January 2021	ICU COVID-19	Air sampler placed approximately 1.3 m above the floor and 2 m from a patient bed, in the center of the room	300 L/min, 10 min
	M4	21 January 2021	ICU intermediate COVID-19	Air sampler placed approximately 1.3 m above the floor and 2 m from a patient bed, in the center of the room	100 L/min, 10 min
	M5	21 January 2021	ICU intermediate COVID-19	Air sampler placed approximately 1.3 m above the floor and 2 m from a patient bed, in the center of the room	200 L/min, 10 min
	M6	21 January 2021	ICU intermediate COVID-19	Air sampler placed approximately 1.3 m above the floor and 2 m from a patient bed, in the center of the room	300 L/min, 10 min
	M7	21 January 2021	Nursing area COVID-19	Air sampler placed approximately 1.3 m above the floor and 2 m from a patient bed, in the center of the room	100 L/min, 10 min
	M8	21 January 2021	Nursing area COVID-19	Air sampler placed approximately 1.3 m above the floor and 2 m from a patient bed, in the center of the room	200 L/min, 10 min
	M9	21 January 2021	Nursing area COVID-19	Air sampler placed approximately 1.3 m above the floor and 2 m from a patient bed, in the center of the room	300 L/min, 10 min
	M10	27 January 2021	Respiratory diseases observation room	Air sampler placed approximately 1.3 m above the floor and 2 m from a patient bed, in the center of the room	100 L/min, 10 min
	M11	27 January 2021	Respiratory diseases observation room	Air sampler placed approximately 1.3 m above the floor and 2 m from a patient bed, in the center of the room	200 L/min, 10 min
	M12	27 January 2021	Respiratory diseases observation room	Air sampler placed approximately 1.3 m above the floor and 2 m from a patient bed, in the center of the room	300 L/min, 10 min
	M13	27 January 2021	Respiratory diseases waiting room	Air sampler placed approximately 1.3 m above the floor in the center of the room	100 L/min, 10 min
	M14	27 January 2021	Respiratory diseases waiting room	Air sampler placed approximately 1.3 m above the floor in the center of the room	200 L/min, 10 min
	M15	27 January 2021	Respiratory diseases waiting room	Air sampler placed approximately 1.3 m above the floor in the center of the room	300 L/min, 10 min
	M16	27 January 2021	Clinical decision unit	Air sampler placed approximately 1.3 m above the floor and 2 m from a patient bed, in the center of the room	100 L/min, 10 min
	M17	27 January 2021	Clinical decision unit	Air sampler placed approximately 1.3 m above the floor and 2 m from a patient bed, in the center of the room	200 L/min, 10 min
	M18	27 January 2021	Clinical decision unit	Air sampler placed approximately 1.3 m above the floor and 2 m from a patient bed, in the center of the room	300 L/min, 10 min
	M19	2 February 2021	COVID-19 testing room	Air sampler placed approximately 1.3 m above the floor in the center of the room	100 L/min, 10 min
	M20	2 February 2021	COVID-19 testing room	Air sampler placed approximately 1.3 m above the floor in the center of the room	200 L/min, 10 min
	M21	2 February 2021	COVID-19 testing room	Air sampler placed approximately 1.3 m above the floor in the center of the room	300 L/min, 10 min
	M22	2 February 2021	Non-respiratory diseases waiting room	Air sampler placed approximately 1.3 m above the floor in the center of the room	100 L/min, 10 min
	M23	2 February 2021	Non-respiratory diseases waiting room	Air sampler placed approximately 1.3 m above the floor in the center of the room	200 L/min, 10 min
	M24	2 February 2021	Non-respiratory diseases waiting room	Air sampler placed approximately 1.3 m above the floor in the center of the room	300 L/min, 10 min
	M25	2 February 2021	Urgency Care (Recovery area)	Air sampler placed approximately 1.3 m above the floor and 2 m from a patient bed, in the center of the room	100 L/min, 10 min
	M26	2 February 2021	Urgency Care (Recovery area)	Air sampler placed approximately 1.3 m above the floor and 2 m from a patient bed, in the center of the room	200 L/min, 10 min
	M27	2 February 2021	Urgency Care (Recovery area)	Air sampler placed approximately 1.3 m above the floor and 2 m from a patient bed, in the center of the room	300 L/min, 10 min
	M28	11 February 2021	Hospital’s outside entrance atrium	Air sampler placed approximately 1.3 m above the floor in the center of the room	100 L/min, 10 min
	M29	11 February 2021	Hospital’s outside entrance atrium	Air sampler placed approximately 1.3 m above the floor in the center of the room	200 L/min, 10 min
	M30	11 February 2021	Hospital’s outside entrance atrium	Air sampler placed approximately 1.3 m above the floor in the center of the room	300 L/min, 10 min
	M31	11 February 2021	Hospital staff’s cafeteria	Air sampler placed approximately 1.3 m above the floor in the center of the room	100 L/min, 10 min
	M32	11 February 2021	Hospital staff’s cafeteria	Air sampler placed approximately 1.3 m above the floor in the center of the room	200 L/min, 10 min
	M33	11 February 2021	Hospital staff’s cafeteria	Air sampler placed approximately 1.3 m above the floor in the center of the room	300 L/min, 10 min

**Table 3:** Details of the surface samples’ collections for SARS-CoV-2 RNA detection in the Hospital.

Sample ID	Hospital Area	Collection Date	Sample Location
S1	COVID-19 testing room	2 February 2021	Hand sanitizer dispenser
S2			Instruments’ counter
S3			Glove box
S4			Wall of a COVID-19 testing booth
S5			Paper dispenser
S6	Non-respiratory disease waiting room	2 February 2021	Faucet handle
S7			Vending machine (buttons)
S8			Bathroom: flush button
S9			Bathroom: inside doorknob
S10			Bathroom: outside doorknob
S11	Hospital’s outside outside entrance atrium	11 February 2021	Statue (approx. 3 m away from outside entrance)
S12			ATM (buttons)
S13			Fire extinguisher (approx. 3 m away from outside entrance)
S14	Hospital staff’s cafeteria	11 February 2021	Soap dispenser
S15			Faucet handle
S16			Paper dispenser
S17			Table sign on a table *

* Table sign: a sign with precautions to avoid contamination was placed on the table.

**Table 4:** Details of Hospital area, sampling location and viral genome copy numbers of the positive air and surface samples.

	Sample ID	Hospital Area	Sample Location	Copy Number (N1 Gene)	Copy Number (N2 Gene)
Air samples	C1	COVID-19 ICU	Air sampler placed approximately 1.3 m above the floor and 2 m from intubated patients beds, in the center of the room	6000 copies/m^3^	6575 copies/m^3^
	M1			6362.5 copies/m^3^	6662.5 copies/m^3^
Surface samples	S4	COVID-19 testing room	Wall of a COVID-19 testing booth	200.6 copies/cm^2^	No amplification detected
	S6	Non-respiratory disease patients’ waiting room	Faucet handle	179.2 copies/cm^2^	No amplification detected
	S8		Bathroom: flush button	No amplification detected	201.7 copies/cm^2^

## Data Availability

Not applicable.
